# Comparative assessment of nutritional, thermal, rheological and functional properties of nine Australian lupin cultivars

**DOI:** 10.1038/s41598-021-00838-x

**Published:** 2021-11-02

**Authors:** Kishor Mazumder, Biswajit Biswas, Philip G. Kerr, Christopher Blanchard, Afia Nabila, Mimi Golder, Mohammad Gulzarul Aziz, Asgar Farahnaky

**Affiliations:** 1 Department of Pharmacy, Faculty of Biological Science and Technology, Jashore University of Science and Technology, Jashore, 7408 Bangladesh; 2 School of Biomedical Sciences, Charles Sturt University, Wagga Wagga, NSW 2650 Australia; 3 Department of Pharmacy, University of Science and Technology Chittagong, Chittagong, 4202 Bangladesh; 4 Pharmacy Discipline, Life Science School, Khulna University, Khulna, 9208 Bangladesh; 5 Department of Food Technology and Rural Industries, Bangladesh Agricultural University, Mymensingh, 2202 Bangladesh; 6 Biosciences and Food Technology, School of Science, RMIT University, Melbourne, 3083 Australia

**Keywords:** Biophysical chemistry, Natural products

## Abstract

Lupin holds an important place among the legumes and the utilization of lupin as a dietary protein source is an excellent environmentally friendly alternative to animal-based products for human nutrition. In the present study, nutritional, thermal, rheological and functional properties of nine Australian lupin cultivars have been assayed in order to find the most valuable one, both nutritiously and industrially. The set comprised six *Lupinus angustifolius* L. viz., Barlock, Gunyadi, Jenabillup, Jindalee, Jurien, Mandelup and three *Lupinus albus* L. viz., Luxor, Rosetta, WK388 cultivars. The tests included analysis of color, macronutrient and micronutrient composition, pasting, textural and thermal properties, electrophoretic profile of protein isolates, swelling power, water and oil absorption capacity, emulsifying capacity, emulsion stability, creaming stability, foaming capacity and stability of the cultivars’ dehulled seed flours. The results indicated substantial variation in macro and micro-nutritional value as well as satisfactory swelling ability, solubility, surface hydrophobicity, foaming ability, emulsifying capacity and gelation property of lupin flours. Superior nutritional, thermal, rheological and functional potential was demonstrated by the *L. albus* cultivars compared to the *L. angustifolius* cultivars with the exception of Mandelup.

## Introduction

Scientists and policy makers around the world are currently seeking sources of sufficient amount of dietary proteins to meet the deficiencies. At the present time, a person in developed countries consume about 103 g of protein per day while in developing countries, average protein intake is below 76 g/capita/day^1^. Animal-based proteins are fulfilling a substantial proportion of dietary protein needs for animal feed and direct human consumption. However, from the financial and ethical perspectives, there is a necessity to develop sustainable plant based protein production^2^. There’s a need to discover plant protein sources for our day to day dietary requirements as the consumption of animal-based products relies on rigorous livestock farming which is environmentally detrimental and depletes natural resources^3,4^. Moreover, high intake of red and processed meat are considered as risk factors for the development of diabetes mellitus, cardiovascular diseases and even some cancers^5,6^. Hence, plant-based dietary protein consumption can be an effective way of maintaining a healthy diet and mitigating negative environmental impact. Attention towards functional foods, i.e. foods with bio-functional benefits beyond their energy providing nutritional value, has increased because of their potential role in maintaining human health and wellbeing^7^. By providing essential nutrients and what are commonly called ‘phytoconstituents’, these foods lessen the risk of developing systemic and chronic diseases in humans^8^.

Lupins belong to the genus *Lupinus*, of the family Fabaceae with four common species, *Lupinus angustifolius* L. (narrow-leaf lupin), *Lupinus albus* L. (white lupin), *Lupinus mutabilis* (tarwi, pearl lupin) and *Lupinus luteus* L. (yellow lupin)^8^. The agricultural history of lupins can be traced back 4000 years. They were first cultivated in the Mediterranean region, followed by the American continent. However, lupin emerged as a modern agricultural crop in Australia and Europe^9^. Among the four species, *L. albus* or the white lupin has always been the most significant in human nutrition, but usage of *L. angustifolius*, or the narrow leaf lupin, is on the rise^8^.

Although wild lupins are bitter tasting and toxic due to the presence of quinolizidine alkaloids (QA), domestication of *L. angustifolius* and *L. albus* has resulted in lower alkaloid content. When the QA content is less than 200 ppm, lupins are considered to be safe for human consumption, and no longer require rigorous treatment to remove the alkaloids before becoming palatable. In addition, de-hulling seeds further reduces the anti-nutritive contents of lupin flours^10,11^. In this instance, lupin is an important grain legume with desirable characteristics. They have low content of anti-nutrients (e.g. phytic acid, lectins and saponins); are free of phytoestrogens and possess good technological properties. Because of their protein content (range 30–50%) they are a potential solution in the search for plant-based dietary protein^8^. Lupin seeds are also a rich source of non-starch polysaccharide (30 to 40%), unsaturated fats (5 to 15%), minerals (Ca, K, P, Mg, Na, Fe, Zn etc.) with limited levels of heavy metals^12,13^. In addition, several studies have reported the bio-functional benefits of lupins viz., hypocholesterolaemic, hypoglycaemic, anti-atherogenic, hypotensive, glycaemic index controlling, antidiabetic and anti-oxidative activities^8,13,14^. Regular consumption of lupins has been demonstrated to counteract insulin resistance and therefore, can alleviate lifestyle disorders like obesity, diabetes and cardiovascular diseases^15^. This is why in recent times, lupin seed flours are being incorporated in processed foods including noodles, pasta, sausages, cereal, cookies, bread, crisps and many others^16^. Moreover, utilization of lupin seeds in traditional fermentation of foods like miso, tofu, sauces, tempe as well as in dairy products like yoghurt, milk without saturated fats has greatly boosted its industrial and market value^15^. However, because of the uncertainty of continuous supply, industrial usage of lupin flours is lessening.

The consumption and production of crops first need vigorous assessment of the ability and feasibility of particular cultivar to provide suitable yield, quality and nutritional value. Moreover, investigation of perceived functional properties is essential to assess the consequences of industrial processing on the crops like mild or harsh mechanical or thermal treatment. This could be due to denaturation or insolubilization of protein isolates by industrial treatment which may have either beneficial or deleterious impact on the nutritive values of the crops^17–19^. Hence in the present study, lupin seed flours, obtained from nine Australian lupin cultivars i.e., six *L. angustifolius* (Barlock, Gunyidi, Jenabillup, Jindalee, Jurien, Mandelup) and three *L. albus* (Luxor, Rosetta, WK388) have been assayed with a view to evaluating their nutritional, thermal, rheological and functional properties. After de-hulling and milling the lupin seeds, the flours obtained were evaluated for color, macronutrient and micronutrient composition, pasting properties, textural and thermal properties and SDS-PAGE profiling of the protein isolates. Among different functional properties, swelling power, water absorption capacity, oil absorption capacity, emulsifying capacity, emulsion stability, creaming stability of emulsion, foaming capacity and finally, foam stability of the Australian lupin flours have been assayed. In short, the present study was designed in with the hope of establishing the significance of lupin as a major food crop in the human diet and finding the most beneficial cultivar in terms of nutritional value and industrial applicability.

## Results

### De-hulling of lupin cultivars

The seed coats of the nine lupin cultivars were separated from the kernels, with weights of both being measured separately. The weights of kernels ranged from 67.42% (Gunyidi) to 73.62% (Rosetta) of totals. Percentage of seed coat was between 24.69 and 15.16. Maximum quantity of seed coat was observed in Gunyidi while the minimum quantity was in WK388. Amount of intermediate material fluctuated between 7.47 and 13.24% (see Supplementary Table S1 online).

### Color evaluation

The average lightness (L*), from black to white; greenness (−) to redness (+) (a*); and blueness (−) to yellowness (+) (b*) values of the nine lupin cultivars are presented in Table 1. Hue angle (h°, presenting property of the color) and chroma (C*), presenting color saturation or intensity, were calculated by using the formulas stated in the methodology^20^. No substantial differences were found in the L* values of the cultivars, with Rosetta (86.68) having the minimum, and Jurien (87.48) the maximum. For the a* values, a wider range of greenness was observed with Luxor (−3.14) and Jenabillup (−2.31). The b* values also showed yellowness, with WK388 (28.59) the lowest, leading to Rosetta and Barlock (both 31.75) at the high end of the spectrum. Although, significant variation in h° was demonstrated, C* was comparable for all cultivars (see Supplementary Fig. S1 online).

**Table 1:** Color evaluation of different lupin cultivars.

Species	Cultivars	L*-Value	a*-Value	b*-Value	C* (Chroma)	h° (Hue angle)
*L. angustifolius*	Barlock	86.83 ± 0.11	− 2.73 ± 0.04^c,g^	31.63 ± 0.15^i^	31.7476	180.67
	Gunyidi	86.93 ± 0.08	− 2.55 ± 0.04^d,g,i^	31.35 ± 0.28^i^	31.45354	183.58
	Jenabillup	87.34 ± 0.06	− 2.31 ± 0.03^a,d,g,h,i^	29.45 ± 0.11	29.54046	174.58
	Jindalee	87.28 ± 0.05	− 2.96 ± 0.02^b,c,e,f^	30.05 ± 0.16	30.19543	178.88
	Jurien	87.48 ± 0.05	− 2.53 ± 0.01^d,g,i^	29.42 ± 0.12	29.52858	180.73
	Mandelup	86.94 ± 0.14	− 2.55 ± 0.02^d,g,i^	31.06 ± 0.09	31.1645	182.46
*L. albus*	Luxor	86.68 ± 0.10	− 3.14 ± 0.02^a,b,c,e,f,h^	30.58 ± 0.30	30.74079	176.92
	Rosetta	86.16 ± 0.06	− 2.66 ± 0.03^c,g^	31.64 ± 0.15^i^	31.75162	181.26
	WK388	87.27 ± 0.12	− 2.96 ± 0.04^b,c,e,f^	28.59 ± 0.08^a,b,h^	28.74282	175.80

Data are means of three replicates with standard deviations (SD). Data within the same column with different superscripts are significantly different, pair-wise comparison by Post Hoc Tukey test (^a^*P* < 0.05 vs. Barlock; ^b^*P* < 0.05 vs. Gunyidi; ^c^*P* < 0.05 vs Jenabillup; ^d^*P* < 0.05 vs. Jindalee; ^e^*P* < 0.05 vs Jurien; ^f^*P* < 0.05 vs. Mandelup; ^g^*P* < 0.05 vs Luxor; ^h^*P* < 0.05 vs Rosetta; ^i^*P* < 0.05 vs WK388). C* and h° are calculated using the means of L*, a*, b* values.

### Macronutrient composition

Protein, fat and fiber compositions of 9 lupin cultivars are presented in Table 2. Protein content (%, w/w) of the cultivars varied between 41.5% (Barlock) and 48.2% (Rosetta). Substantial differences among the fat and fiber content of lupin cultivars were also detected. The lowest fat percentage was demonstrated by Jenabillup (4.4%) and the highest WK388 (11.9%). Soluble fiber content ranged widely from Barlock (2.09%) to Gunyidi (8.24%). Insoluble fiber content varied between 28.9% (Barlock) and 39.55% (Rosetta). Total fiber content was lowest in Rosetta (32.62%) and topped for Gunyidi (42.96%). No substantial differences were observed for dry matter and ash content of the lupin cultivars, with values ranging from 92.6% (Gunyidi) to 94.2% (WK388) for dry matter, and from 2% (Mandelup) to 4% (Gunyidi, Jenabillup, Luxor, WK388) for ash.

**Table 2:** Macronutrient composition of different lupin cultivars.

Species	Cultivars	Dry Matter % (w/w)	Ash % (w/w)	Protein % (w/w)	Fat % (w/w)	Fiber % (w/w)		
						Soluble	Insoluble	Total
*L. angustifolius*	Barlock	93.2 ± 2.24	3 ± 0.1	41.5 ± 2.03^b,d,g,h,i^	4.6 ± 0.66^f,g,h,i^	2.09 ± 0.05^b,c,f,g,h,i^	39.55 ± 2.03^c,d,g,h,i^	41.64 ± 2.07^h^
	Gunyidi	92.6 ± 3.9	4 ± 0.2	46.2 ± 2.39^a,f^	4.7 ± 0.95^f,g,h,i^	8.24 ± 0.21^a,d,e,f,g^	34.72 ± 0.46^h^	42.96 ± 0.25^g,h,i^
	Jenabillup	93.1 ± 0.05	4 ± 0.2	44.6 ± 1.6^h^	4.4 ± 1.08^e,f,g,h,i^	7.65 ± 0.66^a,d,e,f,g,h^	31.4 ± 0.39^a,e^	39.14 ± 1.24^h^
	Jindalee	93.3 ± 0.95	3 ± 0.6	45.7 ± 2.74^a^	4.7 ± 1.49^f,g,h,i^	3.72 ± 0.15^b,c,i^	33.78 ± 2.39^a^	37.5 ± 2.24
	Jurien	92.9 ± 0.41	3 ± 0.05	44.5 ± 1.12	5.8 ± 0.42^c,f,g,h,i^	3.00 ± 0.95^b,c,g,h^	37.24 ± 0.12^c,h,i^	40.24 ± 0.83^h^
	Mandelup	93.4 ± 1.24	2 ± 0.2	42.9 ± 0.13^b,g,h,i^	7.4 ± 0.46^a,b,c,d,e,g,h,i^	4.84 ± 0.41^a,b,c,i^	34.6 ± 2.30^h^	39.44 ± 1.90^h^
*L. albus*	Luxor	94.1 ± 0.66	4 ± 0.7	47.3 ± 2.3^a,f,h^	10.9 ± 0.7^a,b,c,d,e,f^	5.10 ± 0.59^a,b,c,e^	31.9 ± 0.48^a^	37.00 ± 0.12^b^
	Rosetta	93.5 ± 0.41	3 ± 0.2	48.2 ± 3.9^a,c,e,f^	11.7 ± 0.05^a,b,c,d,e,f^	3.17 ± 0.13^b,c,g,h,i^	28.9 ± 0.64^a,b,e,f^	32.62 ± 0.78^a,b,c,e,f^
	WK388	94.2 ± 0.05	4 ± 0.6	46.5 ± 0.95^a,f^	11.9 ± 0.2^a,b,c,d,e,f^	6.64 ± 0.39^a,d,e^	30 ± 1.60^a,e^	36.64 ± 1.21^b^

Data are means of three replicates with standard deviations (SD). Data within the same column with different superscripts are significantly different, pair-wise comparison by Post Hoc Tukey test (^a^*P* < 0.05 vs. Barlock; ^b^*P* < 0.05 vs. Gunyidi; ^c^*P* < 0.05 vs Jenabillup; ^d^*P* < 0.05 vs. Jindalee; ^e^*P* < 0.05 vs Jurien; ^f^*P* < 0.05 vs. Mandelup; ^g^*P* < 0.05 vs Luxor; ^h^*P* < 0.05 vs Rosetta; ^i^*P* < 0.05 vs WK388).

### Microelemental composition

Results for the analyses of the 9 lupin cultivars are presented in Table 3. All cultivars demonstrated the presence of the nutritionally important major minerals with highest levels for Na, Mg, P, K and Ca. The trace elements Mn, Fe and Zn were found at generally lower levels than the major minerals. The nutritionally important Co and Se and toxic Cd and Pb were absent from the lupin flours. The presence of Hg was found only in Jenabillup (0.01 mg/kg). Among the major minerals, K was most abundant, with values ranging between 7054.58 mg/kg (Jindalee) and 8697.9 mg/kg (Jenabillup). Phosphorus was the second most abundant element, ranging from 3433.91 mg/kg (Rosetta) to 4327.81 mg/kg (WK388). Magnesium content was between 1307 mg/kg (Barlock) and 1896.92 mg/kg (WK388). The values for Ca were found to be between 590 mg/kg (Barlock) and 1678.43 mg/kg (WK388). The Na content ranged from 176.33 mg/kg (Jindalee) to 456.79 mg/kg (WK388). For the trace element Mn, the *L. albus* cultivars had the highest content - WK388 (688.58 mg/kg), Rosetta (796.25 mg/kg) and Luxor (1059.96 mg/kg). For the six *L. angustifolius* cultivars, the levels of Mn were considerably lower and found to be between 21.64 and 27.31 mg/kg. In the case of Fe, no substantial differences were observed, and the values fluctuated between 21.67 mg/kg (Luxor) and 30.6 mg/kg (Barlock). For Zn, comparable quantities were observed for all, from 25.03 mg/kg (Jurien) to 31.76 mg/kg (Luxor) with the outlier being WK388 (41.97 mg/kg). While considering the quantity of Cu, the values skyrocketed for Jenabillup (42.59 mg/kg). Finally, all the lupin cultivars displayed a little quantity of Mo with values from 0.23 mg/kg (Jurien) to 3.2 mg/kg (Luxor).

**Table 3:** Microelemental composition of different lupin cultivars.

Species	*L. angustifolius*						*L. albus*			RDI, ESADI & PTI
Elements	Barlock	Gunyidi	Jenabillup	Jindalee	Jurien	Mandelup	Luxor	Rosetta	WK 388	
Na	250 ± 19.80^i^	229.45 ± 28.99^i^	225.78 ± 13.70^i^	176.33 ± 13.71h^,i^	225.67 ± 14.12^i^	225.24 ± 22.92^i^	229.06 ± 15.78^i^	269.76 ± 46.37^d,i^	456.79 ± 11.74^a,b,c,d,e,f,g,h,i^	1100–3300^1^
Mg	1307 ± 94.75^c,e,g,h,i^	1466 ± 17.17^g,i^	1577.74 ± 8.93^a,f,i^	1442.61 ± 1.53^g,i^	1495.38 ± 29.82^a,i^	1398.20 ± 30.76^c,g,i^	1647.81 ± 44.91^a,b,d,f,i^	1546.27 ± 46.00^a,i^	1896.92 ± 35.92^a,b,c,d,e.f,g,h,i^	300–350^2^
P	4012 ± 263.04^d,h^	4165 ± 67.78^d,f,h^	4292.49 ± 31.87^d,g.h^	3535.68 ± 16.57^a,b,c,e,i^	3999.82 ± 52.12^d,h^	3614.76 ± 67.06^b,c,i^	3862.53 ± 102.32^c.h,i^	3433.91 ± 76.99^a,b,c,e,g,i^	4327.81 ± 70.38^d,f,g,h^	800^2^
K	7610 ± 615.89^c^	8150.97 ± 16.68^d^	8697.90 ± 41.28^a,d,f,h,i^	7054.58 ± 44.28^b,c^	8047.46 ± 153.75	7482.97 ± 205.30^c^	8014.98 ± 224.21	7544.57 ± 281.86^c^	7210.67 ± 87.26^c^	1875–5625^1^
Ca	590 ± 24.04^c,d,e,f,g,h,i^	616.99 ± 12.54^c,d,e,f,g,h,i^	863.24 ± 7.23^a,b,d,e,f,g,h,i^	716.62 ± 3.90^a,b,c,g,h,i^	692.26 ± 12.42^a,b,c,g,h,i^	750.21 ± 10.97^a,b,c,g,h,i^	1222.36 ± 27.50^a,b,c,d,e,f,h,i^	1446.48 ± 27.93^a,b,c,d,e,f,g,i^	1678.43 ± 25.08^a,b,c,d,e,f,g,h,i^	800^2^
Mn	22.7 ± 1.41^g,h,i^	25.26 ± 0.46^g,h,i^	21.64 ± 0.23^g,h,i^	25.34 ± 0.43^g,h,i^	27.15 ± 0.51^g,h,i^	27.31 ± 0.42^g,h,i^	1059.96 ± 4.34^a,b,c,d,e,f,h,i^	796.25 ± 21.58^a,b,c,d,e,f,g,i^	688.58 ± 9.19^a,b,c,d,e,f,g,h^	2.5–5.0^1^
Fe	30.6 ± 2.40	27.77 ± 2.06	28.27 ± 2.08	26.62 ± 0.56	25.97 ± 0.65	31.27 ± 5.60	21.67 ± 1.22	22.13 ± 2.49	30.11 ± 0.45	10–18^2^
Co	n/d	n/d	n/d	n/d	n/d	n/d	n/d	n/d	n/d	0.003^3^
Cu	3.18 ± 0.28^c^	3.02 ± 0.11^c^	42.59 ± 0.46^a,b,d,e,g,h,i^	3.02 ± 0.04^c^	3.97 ± 0.90^c^	20.15 ± 23.38	5.35 ± 0.10^c^	5.40 ± 0.10^c^	5.87 ± 0.05^c^	2—3^1^
Zn	28.05 ± 2.05^g,i^	28.26 ± 0.53^g,i^	29.25 ± 0.01^e,i^	27.41 ± 0.14^g,i^	25.03 ± 0.58^c,g,h,i^	26.32 ± 0.83^g,i^	31.76 ± 0.57^a,b,d,e,f,i^	28.65 ± 0.81^e,i^	41.97 ± 0.57^a,b,c,d,e,f,g,h,i^	15^2^
Se	n/d	n/d	n/d	n/d	n/d	n/d	n/d	n/d	n/d	0.05–0.2^1^
Mo	1.07 ± 0.92^g^	0.57 ± 0.13^g,h^	1.10 ± 0.12^g^	0.88 ± 0.16^g,h^	0.23 ± 0.04^g,h,i^	0.62 ± 0.06^g,h^	3.20 ± 0.03^a,b,c,d,e,f,i^	2.22 ± 0.27^b,d,e,f^	1.71 ± 0.19^e,g^	0.15–0.5^1^
Cd	n/d	n/d	n/d	n/d	n/d	n/d	n/d	n/d	n/d	0.001^4^
Hg	n/d	n/d	0.01 ± 0.41	n/d	n/d	n/d	n/d	n/d	n/d	0.0007^4^
Pb	n/d	n/d	n/d	n/d	n/d	n/d	n/d	n/d	n/d	0.007^4^

Data are means of three replicates with standard deviations (SD) and is expressed as mg/kg dry weight; n/d (not detected, below the detection level). Data within the same row with different superscripts are significantly different, pair-wise comparison by Post Hoc Tukey test (^a^*P* < 0.05 vs. Barlock; ^b^*P* < 0.05 vs. Gunyidi; ^c^*P* < 0.05 vs Jenabillup; ^d^*P* < 0.05 vs. Jindalee; ^e^*P* < 0.05 vs Jurien; ^f^*P* < 0.05 vs. Mandelup; ^g^*P* < 0.05 vs Luxor; ^h^*P* < 0.05 vs Rosetta; ^i^*P* < 0.05 vs WK388). ^1^Estimated Safe and Adequate Dietary intake (ESADI); ^2^ Reference daily Intake (RDI); ^3^ expressed as weight of vitamin B12; ^4^ provisional tolerable intakes (PTI) expressed as mg/kg body weight.

### SDS-PAGE profiling of protein isolates

These can be found as Supplementary Fig. S3 online. From the electrophoretic pattern it can be extrapolated that, β-conglutin, the major globulin component of the lupin cultivars, is formed from 10–12 major types of subunit (15–72 kDa); α-conglutin is composed of four main types of subunit (53, 60, 66, and 70 kDa), as well as a number of minor subunits; γ-conglutin, a minor globulin component, is composed of one main type of subunit (42–43 kDa) containing two polypeptide chains. Moreover, below 80 kDa, the bands of six *L. angustifolius* cultivars were very similar. Jenabillup, Jurin and Mandelup showed few large proteins and peptides. On the other hand, two *L. albus* cultivars, Luxor and Rosetta, demonstrated a total band similarity.

### Pasting properties

**Figure 1:**
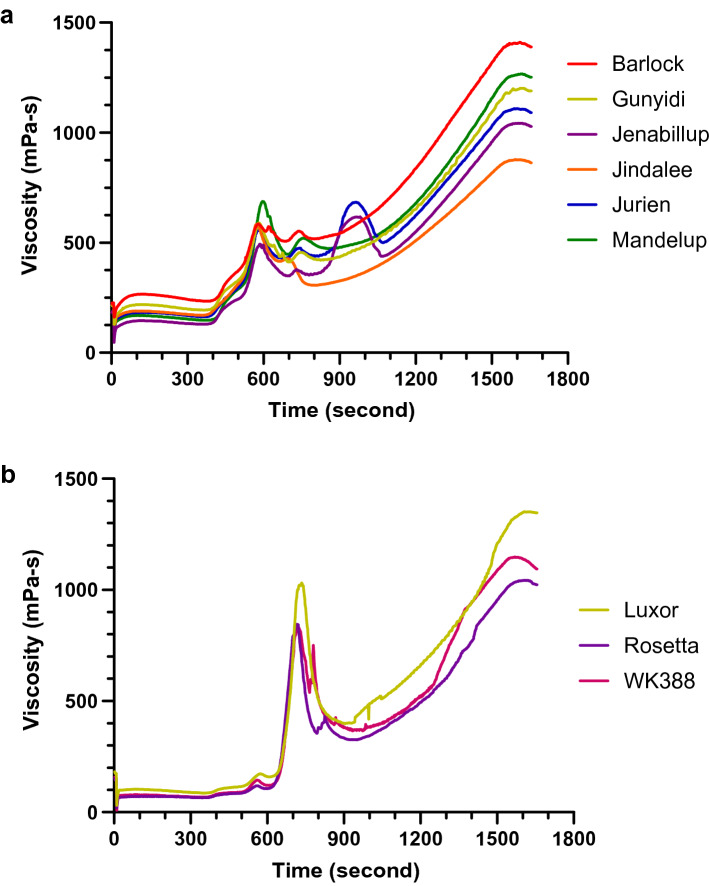
Pasting properties of different lupin cultivars’ flours, analyzed by RVA; where (**a**): RVA pattern of *L. angustifolius* cultivars and (**b**): RVA pattern of *L. albus* cultivars.

Pasting properties of the lupin flours were measured by a Rapid Viscosity Analyzer (RVA) and the results are shown in Fig. 1 and Supplementary Table S2 online. Peak 1 represents the maximum viscosity attained in the heating phase of RVA analysis and the values ranged from 122.5 mPa-s (Rosetta) to 688 mPa-s (Mandelup). The trough, i.e. minimum, viscosity achieved at maximum temperature, fluctuated between 327.5 mPa-s (Rosetta) and 512 mPa-s (Barlock). Peak 2, the maximum increase in viscosity during the heating phase of RVA analysis, bottomed out 379.5 mPa-s (Jenabillup) and peaked at 1036 mPa-s (Luxor). Peak 3, the maximum rate of viscosity reduction, was observed at 426.03 mPa-s (Jurien) and 316.2 mPa-s (Jenabillup)^21^. WK388 took the shortest time to achieve the maximum viscosity (4.85 min) while Mandelup took the longest (9.92 min). In regard to the peak 2 time, no substantial variations were observed and values were between 11.54 (Jindalee) and 12.59 min (Mandelup). Both Jurien and Jenabillup took about 16 minutes to reach peak 3. Finally, the final viscosities of lupin cultivars were measured and the values ranged from 863 mPa-s for Jindalee to 1593 mPa-s for Luxor. Compared to starch rich grains such as wheat, rice and corn, lupin flours had substantially lower RVA viscosity levels that can be accounted for by the virtual absence of starch in lupin flours.

### Textural properties

**Figure 2:**
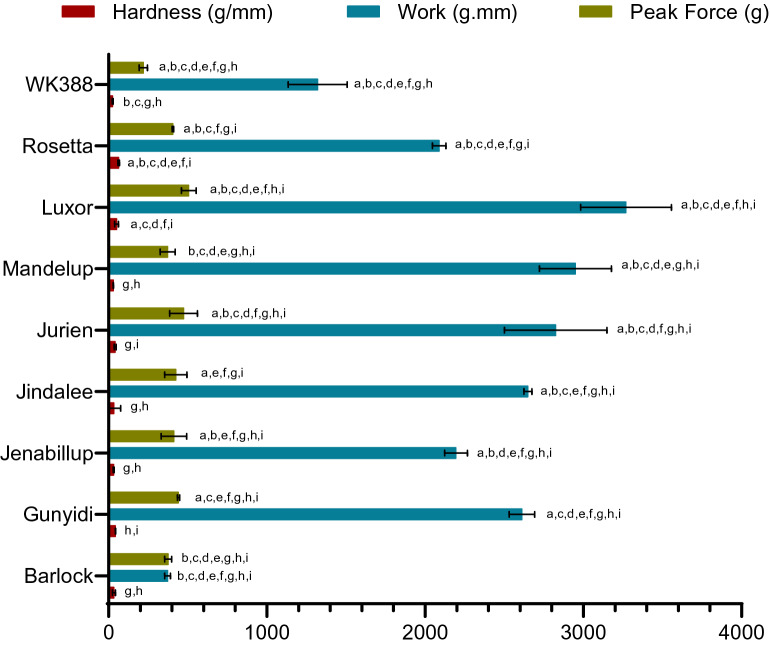
Texture analysis parameters of lupin flours of different cultivars. Data are means of three replicates with standard deviations (SD). Pair-wise comparison by Post Hoc Tukey test, bars with alphabetic characters demonstrate significant differences (^a^ P < 0.05 vs. Barlock; ^b^ P < 0.05 vs. Gunyidi; ^c^ P < 0.05 vs Jenabillup; ^d^ P < 0.05 vs. Jindalee; ^e^ P < 0.05 vs Jurien; ^f^ P < 0.05 vs. Mandelup; ^g^ P < 0.05 vs Luxor; ^h^ P < 0.05 vs Rosetta; ^i^ P < 0.05 vs WK388).

The textural properties of lupin cultivars were determined by a texture analyzer using the samples prepared for RVA and left overnight. Results are presented in Fig. 2. The parameters of textural properties, i.e., hardness, work and peak force, varied significantly among the cultivars (see Supplementary Table S3 online). The variation in hardness was within an order of magnitude, being lowest for WK388 (26.71 g/mm) and highest for Rosetta (63.68 g/mm). The work required for penetration of gels formed varied over an order of magnitude with that of Barlock requiring the minimum amount of work (373.41 g.mm), while the Luxor gel required the maximum work for penetration (3269.63 g.mm). The peak force required during punching also varied within and order of magnitude, and was lowest for WK388 (220.12 g) and topped for Luxor (506.59 g).

### Thermal properties

**Figure 3:**
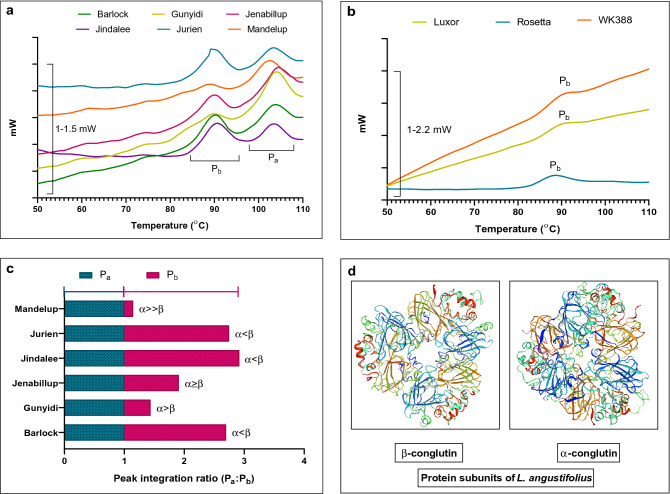
DSC analysis of lupin flours of different cultivars showing presence of β- and α-conglutin; where (**a**): shows the thermograms for the *L. angustifolius* cultivars, (**b**): shows the thermograms for the *L. albus* cultivars, (**c**): bar graph depicting the endothermic peak integration ratio (P_a_:P_b_), where P_a_ is the integration of α-conglutin and P_b_ is the integration of β -conglutin. Here, (**d**) is presenting the structures of β- and α-conglutin proteins obtained using the SWISS-MODEL. DSC analysis was carried out in triplicates.

While analyzing the thermal properties of lupin cultivars using differential scanning calorimetry (DSC), the onset temperature of heat absorption (T_0_), the denaturation or peak temperature (T_d_), conclusion or end-set temperature (T_e_) and the transition heat or denaturation enthalpy (∆H) of raw flour were recorded and presented in Fig. 3 and Table 4 (thermograms of protein isolates can be found as Supplementary Fig. S2 online)^22^. In the thermograms, two endothermic peaks were observed for the *L. angustifolius* cultivars, which indicated the two major globulins protein classes—the vicilins (β-conglutin) and the legumins (α-conglutin)^8^. Whereas for the *L. albus* cultivars, only a single peak corresponding to the β-conglutins was observed. Significant differences in the T_o_, T_d_, T_e_, ∆H values of the β-conglutin peak were observed for all cultivars. The lowest values of T_o_, T_d_, T_e_ are demonstrated by Gunyidi (79.61, 88.24 and 93.15 °C, respectively) with the highest being observed for both Jindalee and Mandelup (84.69, 90 and 95.05 °C, respectively). Gunyidi has the lowest denaturation enthalpy (0.92 J/g), with Rosetta having the highest (2.74 J/g). Endothermic peaks for α-conglutin were observed only for the 6 *L. angustifolius* cultivars with no substantial differences in the T_o_, T_d_ and T_e_ values being found. However, significant variations in the denaturation enthalpy were detected, with the values ranging from 0.72 J/g (Barlock) to 2.12 J/g (Gunyidi). Peak integration ratios (Fig. 3c) of the 6 lupin cultivars were also calculated from which, variations in the quantity of α- and β- conglutins were determined.

**Table 4:** Thermal analysis of different lupin cultivars.

Species	Cultivars	Peak-1 (β-Conglutin Endothermic Peak)				Peak-2 (α-Conglutin Endothermic Peak)			
		∆H (J/g)	T_0_ (°C )	T_d_ (°C )	T_e_ (°C )	∆H (J/g)	T_0_ (ºC)	T_d_ (°C)	T_e_ (°C)
*L. angustifolius*	Barlock	1.38 ± 0.09^g,h,i^	84.43 ± 0.22 ^b,c,g,h,i^	89.59 ± 0.16^h^	93.99 ± 0.33^h,i^	0.72 ± 0.88^b,c^	97.79 ± 0.76	103.06 ± 0.08	107.53 ± 0.14
	Gunyidi	0.92 ± 0.08^d,e,f,g,h,i^	79.61 ± 0.21^a,c,d,e,f,g,h,i^	88.86 ± 0.26^d,f^	93.15 ± 0.30^d,f,g,h,i^	2.12 ± 0.32^a,c,d,e,f^	97.84 ± 0.33	103.18 ± 0.27	108.01 ± 0.34
	Jenabillup	1.26 ± 0.08^g,h,i^	83.26 ± 0.23^a,b,d,e,f,g,i^	89.20 ± 0.01	93.31 ± 0.03^d,f,g,h,i^	1.38 ± 0.01^a,b^	98.28 ± 0.30	103.33 ± 0.47	108.26 ± 0.36^e^
	Jindalee	1.72 ± 0.11^b,h,i^	84.69 ± 0.07^b,c,g,h,i^	90.07 ± 0.01^b,h^	95.05 ± 0.15^b,c^	0.90 ± 0.04^b^	98.98 ± 0.0	103.21 ± 0.23	107.44 ± 0.13
	Jurien	1.46 ± 0.07^b,h,i^	84.66 ± 0.40^b,c,g,h,i^	89.94 ± 0.18^h^	94.62 ± 0.74	0.84 ± 0.06^b^	98.76 ± 0.25	103.09 ± 0.06	107.32 ± 0.05^c^
	Mandelup	1.72 ± 0.11^b,h,i^	84.69 ± 0.07^b,c,g,h,i^	90.01 ± 0.01^b,h^	95.05 ± 0.15^b,c^	1.51 ± 0.03^b^	98.98 ± 0.0	103.21 ± 0.23	107.43 ± 0.13
*L. albus*	Luxor	1.93 ± 0.02^a,b,c,h^	81.55 ± 0.07^a,b,c,d,e,f^	89.16 ± 0.14	95.48 ± 0.28^b,c^	n/d			
	Rosetta	2.74 ± 0.06^a,b,c,d,e,f,g^	82.60 ± 0.42^a,b,d,e,f,i^	88.24 ± 0.34^a,d,e,f,i^	95.91 ± 0.42^a,b,c^	n/d			
	WK388	2.32 ± 0.28^a,b,c,d,e,f,g^	81.34 ± 0.42^a,b,c,d,e,f,h^	89.77 ± 0.71^h^	96.11 ± 0.57^a,b,c^	n/d			

Data are means of three replicates with standard deviations (SD); n/d (Not detected). Data within the same column with different superscripts are significantly different, pair-wise comparison by Post Hoc Tukey test (^a^*P* < 0.05 vs. Barlock; ^b^*P* < 0.05 vs. Gunyidi; ^c^*P* < 0.05 vs Jenabillup; ^d^*P* < 0.05 vs. Jindalee; ^e^*P* < 0.05 vs Jurien; ^f^*P* < 0.05 vs. Mandelup; ^g^*P* < 0.05 vs Luxor; ^h^*P* < 0.05 vs Rosetta; ^i^*P* < 0.05 vs WK388).

### Functional properties of lupin cultivars

Results for these data are given in Table 5. In evaluating the swelling power, Rosetta demonstrated the lowest swelling capacity (3.37 g/g) while Jurien the highest (4.01 g/g). Gunyidi (4 g/g) had a swelling ability close to Jurien. Values for Barlock, Jenabillup, jindalee and Mandelup were comparable, ranging from 3.84 to 3.89 g/g. Both the water and oil absorption capacity was maximum in Jurien (4.4 and 2.23 mL/g, respectively). Mandelup had the least oil absorption capacity (1.7 mL/g) but its water absorption capacity (4.2 mL/g) was comparable to that of Jurien. WK388 showed the least water absorption capacity (1.73 mL/g). Significant differences in the emulsifying capacity and emulsion stability were demonstrated by the lupin cultivars. The highest emulsifying capacity and emulsion stability was 25.94% (for Luxor) and 43.41% (for Mandelup), respectively. However, the least emulsifying capacity and emulsion stability was 19.36 and 20.76%, respectively (for Barlock). For the creaming stability of emulsions, significant variations were observed and the values fluctuated between 35.77% (Mandelup) and 43.53% (Jurien and Luxor). The foaming capacity and foam stability of lupin flours were also measured and the data exhibited significant differences with values ranging from 11.32 (Barlock) to 20.75% (Mandelup) and 5.66 (Barlock) to 13.84% (Mandelup), respectively.

**Table 5:** Functional properties of different lupin cultivars.

Species	Cultivars	Swelling power (g/g) ± SD	Water absorption capacity (mL/g) ± SD	Oil absorption capacity (mL/g) ± SE	Emulsifying capacity (%) ± SD	Emulsion stability (%) ± SD	Creaming stability of emulsion (%) ± SD	Foaming capacity (%) ± SD	Foam stability (%) ± SD
*L. angustifolius*	Barlock	3.89 ± 0.08^g^	4.33 ± 0.12^b,c,d,g,h,i^	1.8 ± 0.35^c,d,e,h,i^	19.36 ± 0.22^f,g,i^	20.76 ± 0.66^b,c,d,e,f,g,h,i^	40.28 ± 1.44^b,c,d,e,f,g,h,i^	11.32 ± 8.22^c,d,e,f,g,h,i^	5.66 ± 8.21^c,d,e,f,g,h,i^
	Gunyidi	4.0 ± 0.13^g,h^	3.13 ± 0.42^a,c,e,f,h,i^	1.87 ± 0.12^d,e^	21.66 ± 0.23^f,g^	41.41 ± 1.23^a,c,d,f,g^	35.89 ± 7.64^a,d,e,g,i^	13.21 ± 3.27^c,e,f,g,h,i^	6.28 ± 2.88^d,e,f,g,h,i^
	Jenabillup	3.87 ± 0.07	2.33 ± 0.42^a,b,d,e,f,g,i^	2.13 ± 0.12^a,f^	21.02 ± 0.97^f,g^	36.92 ± 3.15^a,b,e,f,h,i^	36.92 ± 3.15^a,d,e,f,g,i^	16.35 ± 1.09^a,b,f^	8.17 ± 2.88^a,f,i^
	Jindalee	3.87 ± 0.24	2.89 ± 0.58^a,c,e,f,g,h,i^	2.2 ± 0.35^a,b,f,g^	21.28 ± 0.59^f,g^	37.46 ± 3.47^a,b,e,f,h,i^	43.53 ± 2.89^a,b,c,f,h^	15.08 ± 4.10^a,e,f^	9.43 ± 5.66^a,b,f^
	Jurien	4.01 ± 0.11^g,h^	4.4 ± 0.72^b,c,d,g,h,i^	2.23 ± 0.31^a,b,f,g^	20.64 ± 0.59^f,g^	41.28 ± 1.97^a,c,d,f,g^	43.21 ± 0.59^a,b,c,f,h^	17.61 ± 4.09^a,b,d,f^	10.06 ± 1.09^a,b,f^
	Mandelup	3.84 ± 0.23	4.2 ± 0.72^b,c,d,g,h,i^	1.7 ± 0.23^c,d,e,h,i^	24.61 ± 1.76^a,b,c,d,e,h^	43.41 ± 1.77^a,b,c,d,e,g,h,i^	35.77 ± 3.85^a,c,d,e,g^	20.75 ± 1.89^a,b,c,d,e,g,h,i^	13.84 ± 2.88^a,b,c,d,e,g,h,i^
*L. albus*	Luxor	3.35 ± 0.21^a,b,e^	3.33 ± 0.31^a,c,d,e,f,h,i^	1.87 ± 0.46^d,e,f^	25.94 ± 1.13^a,b,c,d,e,h,i^	37.46 ± 3.47^a,b,e,f,h,i^	43.53 ± 2.89^a,b,c,f,h,i^	16.46 ± 4.75^a,b,f^	8.84 ± 6.01^a,b,f,i^
	Rosetta	3.37 ± 0.19^b,e^	2.2 ± 0.2^a,b,d,e,f,g,i^	2.13 ± 0.23^a,f^	21.29 ± 0.06^f,g^	41.41 ± 1.23^a,c,d,f,g^	35.89 ± 3.49^a,d,e,g,i^	16.40 ± 4.36^a,b,f^	10.06 ± 3.93^a,b,f^
	WK388	3.55 ± 0.04	1.73 ± 0.23^a,b,c,d,e,f,g,h^	2.13 ± 0.12^a^	22.64 ± 1.43^a,g^	41.28 ± 1.97^a,c,d,f,g^	43.21 ± 0.59^a,b,c,f,h^	16.35 ± 2.89^a,b,f^	11.32 ± 1.89^a,b,c,f,g^

Data are means of three replicates with standard deviations (SD). Data within the same column with different superscripts are significantly different, pair-wise comparison by Post Hoc Tukey test (^a^*P* < 0.05 vs. Barlock; ^b^*P* < 0.05 vs. Gunyidi; ^c^*P* < 0.05 vs Jenabillup; ^d^*P* < 0.05 vs. Jindalee; ^e^*P* < 0.05 vs Jurien; ^f^*P* < 0.05 vs. Mandelup; ^g^*P* < 0.05 vs Luxor; ^h^*P* < 0.05 vs Rosetta; ^i^*P* < 0.05 vs WK388).

## Discussion

After dehulling the lupin cultivars, the colors of the seed flours were evaluated with a Minolta CT-310 Colorimeter. All flours demonstrated comparable lightness, presented by the L* values. Moreover, all 9 flours of lupin cultivars had a similar greenish-yellow color, confirmed by small negative a* values (ranging from − 2.31 to − 3.14) and large positive b* values (varying between 28.59 and 31.75). The color intensity or chroma, presented by closely related C* values, was also quite similar. For the hue angle (h°), values can vary from 0° (pure red) and 270° (pure blue) where, 90° is pure yellow and 180° is pure green. All the lupin flours had an h° around 180° which is also a representative of their greenish yellow color^20,23^. Hence, the greenish yellow color of all nine flours can be easily be equated. Effectively all cultivars had the same color profile. None really is distinguishable from the other.

Different studies have been conducted to compare the macronutrient composition of lupin with other plant protein sources. Amongst the different legumes, pulses, beans, peas or lentils, lupin has been reported with the highest amount of dietary fiber and protein^2,13^. Our studies are consistent with these reported findings. Lupin macronutrient composition also varies among species according to location and genotype and its protein content has been reported to be within the 30–50% range. Soluble and insoluble fiber content was between 30 to 40% and lipid content ranged from 5 to 15% of dry matter^12,24^. In our study, the lupin flours demonstrated substantial amounts of protein (all over 40%). The *L. albus* cultivars i.e., Luxor, Rosetta and WK388, possess higher protein contents than the six *L. angustifolius* cultivars*.* Plant proteins are often considered to be inferior to animal protein. However, the essential amino acids—histidine, leucine, lysine, isoleucine, threonine, tryptophan, valine—have been reported in both *L. albus* and *L. angustifolius*^25^. This certainly gives lie to the myth of plants being sources of low quality protein. Having such high protein contents, lupins can provide the essential amino acids in considerable abundance, with perhaps the *L. albus* species being the superior protein source. Although the fat content of lupin cultivars is between 4.6 and 11.9%, studies have reported the presence of the essential fatty acids for humans—linoleic (omega-6) and linolenic (omega-3) acids—which have substantial health benefits^13^. Turning into the details, *L. albus* is reported to be comprised of 13.5% saturated fats, 55.4% monounsaturated fats and 31.1% polyunsaturated fats. While for *L. angustifolius*, the saturated fats content is reported to be 15.1% and the unsaturated fats content is 84.9%. The main fatty acids of *L. albus* are palmitic, stearic, oleic, linoleic, linolenic, gadoleic and erucic acids while for *L. angustifolius*, the major fatty acids are linoleic, oleic, palmitic and linolenic acids^15,16,26^.

The total fiber content for each of the cultivars was over 35%. The dietary fiber that helps the natural functioning of colon, constitutes a significant portion of lupin cultivars’ flours. Instead of starch that increases blood glucose level, the major polysaccharide carbohydrates of lupin are hemicellulose, cellulose, pectin, with galactose and arabinose amongst other monosaccharides. This fiber helps to control the glucose level as well as lower blood cholesterol^27^. This makes lupins an attractive source of dietary fiber and therefore a plant food that is worth promoting as a healthy food. Thus we can be assured that the macronutrient compositional analysis of the Australian lupin cultivars clearly indicates their dietary and nutritional value.

Lupin fulfills substantial proportion of dietary vitamin requirements for human with high contents of vitamin B and E. The thiamin, riboflavin and niacin in *L. albus* have already been reported and the values were 3.9, 2.3 and 39.1 mg/kg, respectively^16^. *L. albus* is also reported for its high tocopherol contents, specifically the γ-tocopherol (20.1 mg/100g) and its moderate content of vitamin C (6.48 mg/100g)^28^. *L. angustifolius* on the other hand, has been reported to have 7.12 g/kg of thiamin and 2.36 g/kg of rivoflavin. It also has high γ-tocopherol contents (10.30 g/kg) with substantial amount of α- and β- tocopherol. Vitamin C is reported to be absent here^29^.

Diets deficient in minerals have detrimental effects on human health, and common mineral deficiencies include iron, zinc, magnesium, iodine, calcium, potassium, and phosphorus, which is currently affecting about 3.7 billion people worldwide^30^. Typical diets may not fulfill mineral requirements; hence, fortifications with minerals are warranted. Based on the results of our study, the utilization of lupin to improve the nutritional value of foods can be a cost effective approach to fight the mineral deficiencies^2^. Our microelement evaluation of lupin cultivars demonstrated the presence of the essential major minerals including Mg, K, Ca, P, Na, Fe and Zn which is essential for blood pressure regulation as well as maintaining balance of acid-base and functionality of muscles^31^. The significant differences we observed in the microelement composition among the nine lupin cultivars is undoubtedly due to multiple factors like different environmental conditions, soil characteristics and fertilizer applications. Traces of Hg were found in the flour sample of only one cultivar, viz. Jenabillup, but the concentration was far below the safe margin. Heavy metal contamination can be due to urban development, fertilizer or pesticide application or milling^32^. A substantial difference in the Mn content between two lupin species was observed in our study where the values for *L. angustifolius* ranged from 21.64 to 27.31 mg/kg and for *L. albus*, between 688.58 and 1059.96 mg/kg. This may be attributed to plant genotype, Mn containing fertilization, soil condition and grain processing methods^33^.

After evaluating the nutritional properties of nine lupin cultivars, a comparative model can be obtained by in depth reviewing of the results. All the *L. albus* cultivars i.e., Luxor, Rosetta and WK388, are rich in both macronutrient (protein, fat, and fiber) and macronutrient (Mg, K, Ca, P, Na, Fe, Zn etc.) composition and among these, WK388 demonstrated the most significant nutritional potential with high level of Na, Mg, P, Ca, Zn, Fe, protein and fat content. The nutritional potential of six *L. angustifolius* cultivars is not lagged at any instances, but comparatively at a lower level.

In the SDS-PAGE analysis, major protein bands of the flours were detected within a range of 3.5 to150 kDa. Two major globulin components were observed, one having 10–12 subunits each within a range of 15–72 kDa and another having 4 subunits at 53, 60, 66, 70 kDa. The DSC analysis of lupin flours is consistent with the SDS-PAGE analysis in that it demonstrated the presence of two endothermic peaks for corresponding to major globulin proteins. Subunit predominance was judged on the basis of intensity and band width. These evaluations correspond to previous studies where major subunits of lupin seed proteins were identified within the range of 20 to 150 kDa^34^. Again, a minor globulin component with two polypeptide chains and one main subunit, viz. γ-conglutin, was also identified at 42–43 kDa. This protein is ascribed as possessing a hypoglycemic effect^35^. Finally, a clear disparity in the electrophoretic pattern of SDS-PAGE between the *L. angustifolius* and *L. albus* cultivars highlights the variations in their globulin component.

Pasting properties of starch rich flours depend on the viscosity or swelling potential of starch granules, which is affected by the molecular structure of amylose and amylopectin, their size and content of amylose leaching out into the solution during gelation^36^. In our evaluation using RVA (see Fig. 1), we observed significant differences for maximum viscosity (peak 1), time of maximum viscosity, minimum viscosity at maximum temperature (trough), maximum increase in viscosity (peak 2), the time of maximum increase in viscosity and maximum reduction rate of viscosity (peak 3). The RVA pattern for Jenabillup and Jurien was fairly comparable as both had three peaks. Peak 1 viscosity as well as the time to reach peak 1 was greater for the *L. angustifolius* cultivars compared to the *L. albus* cultivars. Pasting viscosity of three *L. albus* cultivars improved with temperature. Final viscosities of the lupin flours increased gradually, indicating the development of pasting viscosities under experimental conditions. Lupin flours have low starch levels. RVA viscosity developments and changes of lupin flours are therefore, related to hydration behavior of lupin proteins and fiber (in particular soluble fiber) and interaction of these biopolymers with water molecules during heating and mixing in excess water. In our study, due to the low starch content, moderate final viscosities were observed for all the lupin flours, in comparison to RVA profiles of other starch rich flours^37^.

When the lupin flour pastes were assayed, significant variations were observed for the hardness, work and peak shearing force. Rosetta, which had the minimum peak viscosity, demonstrated maximum hardness. Gel hardness of Barlock, Jenabillup, Jindalee and Mandelup was comparable to this. Retrogradation of starch, content of protein, higher content of amylose and crystallization of amylopectin may govern the gel firmness. Presence of high molecular weight polysaccharides usually affects the water absorption or penetration properties of gels. Luxor, who showed maximum increase in viscosity and had fairly high fiber content, required maximum work to penetrate the gel. Water penetration property of Gunyidi, Jindalee, Jurin was almost similar. Finally, the shearing force, a characteristic of rheological property, rigidity, volume fraction and interaction of phases, varied significantly among the lupin flour gels^36,38^. In short, relatively moderate hardness, water penetration capability and peak force of lupin flour gels are attributed to their lower amylose and amylopectin content as well as weak gelling properties of its biopolymers i.e. protein and fiber.

In the analysis of thermal properties, lupin flour was assayed by DSC and flour protein was converted from the native to the denatured state by heating, which in turn, disrupted intramolecular bonding and unfolded protein aggregation^22^. In the present study, the onset temperature (T_0_), the denaturation temperature (T_d_), the end-set temperature (T_e_) and the denaturation enthalpy (∆H) was measured for two endothermic peaks representing two globulin proteins—β-conglutin and α-conglutin. Both endothermic peaks were present in the six *L. angustifolius* cultivars while the three *L. albus* ones demonstrated only β-conglutin endothermic peak. In considering the β-conglutin endothermic peak, less T_0_ and T_d_ but higher ∆H was observed for *L. albus* cultivars compared to *L. angustifolius* ones. Significant differences among cultivars in the ∆H, T_0_, T_d_ and T_e_ was observed. However, for the α-conglutin endothermic peak, comparable T_0_ and T_d_ with small variations in the ∆H and T_e_ was detected. The differences in the T_0_, T_d_, T_e_ and ∆H values for the different lupin cultivars may be attributed to their varying types of globulin content and heating temperature because the unfolding of protein is influenced by intermolecular contacts^39^. The values are representative of a combination of the endothermic and exothermic reactions like hydrogen bond disruption and break up of hydrophobic interactions, and so, the DSC curve is a ‘fingerprint’ of measured protein fractions^40^. In our assay, DSC analysis was conducted on raw flour sample in the presence of excess water, without isolating the proteins and when compared with the thermograms of isolated β- and α-conglutin, negligible disparity in peak intensity were observed (see Supplementary Fig. S2 online.). Moreover, the peak integration ratio of β- and α-conglutin endothermic peaks, denaturation temperature and denaturation enthalpy can be utilized for qualitative evaluations like identification of different cultivars, conformational changes of lupin proteins, protein stability assay with temperature in future^41^.

Significant differences among the tested functional properties of lupin flours have been detected. Swelling power of flour is a representative of amylose and amylopectin content where amylose acts as an inhibitor of swelling^42^. Moreover, the capacity of the flour to retain water after gelatinization through breakdown of existing hydrogen bond between starch and generation of new bonds with water, also affects the swelling ability^43^. Hence, cultivars with greater swelling power namely, Barlock, Gunyidi, Jurien, Mandelup also had greater water absorption capacity. Oil absorption capacity depends on the lipophilic nature of lupin proteins where non-polar side chains of amino acids bind to the hydrophobic chains of fats^34^. Emulsifying capacity is directly affected by protein solubility, pH, protein concentration and lipophilicity^44^. The creaming behavior of emulsion, however, is dependent upon the viscosity and globule size^45^. Finally, the foaming properties are affected by the pH and protein concentration where foaming capacity and foam stability decrease at the isoelectric point and increase with protein concentration^46^. While comparing the functional properties of the cultivars, it can be concluded that, Mandelup exhibited the best foaming capacity as well as substantial swelling power, water absorption capacity, emulsifying capacity, emulsion stability and foam stability. Among the others, Luxor displayed highest foaming and emulsifying capacity as well as creaming stability of emulsion, Rosetta and WK388 displayed significant oil absorption capacity, foaming capacity, emulsion stability, foam stability and creaming stability of emulsion. Functional potential of Barlock, Gunyidi, Jenabillup, Jindalee and Jurien were moderate.

To sum up, the *L. angustifolius* and *L. albus* cultivars have a valuable macronutrient and microelement profile with substantial health advantages including improvement of satiety, stabilization of blood glucose level, enhancement of bowel function, reduction of blood cholesterol level and blood pressure. Moreover, their favorable phytochemical profiles, which include wide range of polyphenols (p-hydroxybenzoic acid, protocatechuic acid, gallic acid, coumaric acid, catechin and epicatechin etc.), flavonoids, isoflavones, tannins, saponins,, conglutin proteins etc., also have been reported for anti-inflammatory, antioxidative, hypoglycemic, anticancer effects^2,13^. After comparing nine cultivars, nutritional, thermal, rheological and functional potential are found to be comparatively greater in *L. albus*. However, Mandelup, a *L. angustifolius* cultivar, demonstrated the most substantial functionality amongst all. The present study has undoubtedly proven lupin’s worth over other pulses and therefore, will be beneficial in potentiating lupin’s utilization as an alternative protein source to the growing global population. The thermal, rheological and functional properties also signify its utilization in fermented foods, in dairy products, and in nutritionally poor foods as plant-derived meat equivalents. Thus, increased lupin incorporation in our dietary food products can in turn, boost up the functionality and health benefits. However, the study would have benefitted from an analysis of the major vitamins present in the cultivars as well as an investigation of their lipid and carbohydrate compositions. Moreover, the underlying reasons for the difference between nutritional and functional properties among the cultivars have not been assessed properly. Although information on the vitamin contents, lipid compositions and the causes of variations between the two lupin species have been provided from existing literatures, further works on this are recommended. In addition, due to some technical difficulties, for instance absence of Cr standards, we failed to carry out some tests. These assessments in future will make the work absolute and will stimulate the utilization of lupin as plant oriented protein source in human diet.

## Methods

### Standards, chemicals, reagents and methods

Standards and general laboratory reagents were purchased from Sigma-Aldrich (Gillingham, England) and Fisher Scientific UK Ltd. (Loughborough, England). Chemicals used for Inductively Coupled Plasma Optical Emission Spectrometry (ICP-OES) analysis were nitric acid (TraceSelect Ultra) from Fluka (Gillingham, England), hydrochloric acid (30%, Ultrapure) from Merck (Darmstadt, Germany), and deionized water from Millipore (Bedford, USA). All single element standards for ICP-OES were purchased from Inorganic Ventures (Christiansburg, USA). All experiments included in this manuscript were complied with local and national regulations/guidelines and approved by the appropriate authority of the Charles Sturt University.

### Collection and dehulling of lupin cultivars

Nine lupin cultivars’ seeds, six *L. angustifolius* i.e., Barlock, Gunyadi, Jenabillup, Jindalee, Jurien, Mandelup and three *L. albus* cultivars i.e., Luxor, Rosetta, WK388, were collected from New South Wales Department of Primary Industries (NSW DPI), Wagga Wagga Agricultural Institute, Australia. The seeds were mechanically dehulled with a dehulling machine available at DPI Research Center. After dehulling, the seed coats were separated from kernels by sieving. The kernels were then finely ground to pass through a 300 μm sieve using milling machine available at the Wine Research Center of Charles Sturt University, NSW, Australia. Initial weight, kernel weight, weight of seed coat and weight of the intermediate materials were separately measured. Later, the kernel flours were stored in cold room.

### Color evaluation

Colors of the lupin cultivars were represented in terms of chroma or color intensity [C* = (a*^2^+b*^2^)^1/2^] and hue angle [h°= 180 + tan^−1^ (b*/a*)] using a Minolta CT-310 Colorimeter, with illuminant D_65_ and 10° observer angle^47^. In the CIELab color model, a* represents the red (+) to green (−) axis, b* the yellow (+) to blue (−) axis and L* represents lightness. These parameters were measured against a white background with the flour samples fitted in a container (height: 2 mm; diameter: 20 mm), the determinants were carried out in triplicates^48,49^.

### Macronutrient composition

The macronutrient composition i.e., dry matter, ash, protein, fat and soluble or insoluble fiber content of the lupin flours were determined by employing routine proximate analytical procedures by using the following methods: dry and grind in dry matter (Reuter & Robinson 2.E.3; 2.E.4 Method ID: LMOP 2-1100), dry matter digestibility (Wet chemistry; AFIA Method 1.7R Method ID: LMOP 2-1128 2), crude protein (DUMAS Combustion Method; AOAC 990.03, Method ID: LMOP 2-1112), crude fat by hexane soxhlet extract (Wet chemistry—CSL, Method ID: LMOP 2-1122), acid detergent fiber (CSL, Method ID: LMOP 2-1108), neutral detergent fiber (Wet chemistry; CSL, Method ID: LMOP 2-1107), calculation of metabolisable energy (AFIA Method 2.2R; Based on PC DOMD Method ID: LMOP 2-1124). All the experiments were conducted in triplicates at DPI, Wagga Wagga Agricultural Institute^50^.

### Microelemental composition

Analysis of the microelemental composition of lupin samples was done according to the method described by Multari et al. with minor modifications^2^. Samples of different lupin flours (0.5 g) were transferred to a 250 mL tall form beaker. Nitric acid (65% v/v, 10 mL) solution was added, the beaker was covered with a watch glass and the samples were heated to ‘reflux’ on low heat for 3–4 h on a hotplate. To avoid drying out, more nitric acid was added to the sample if the volume was too low. When all brown fumes were dissipated and a light colored clear solution remained, sample digestion was considered to be complete. The samples were removed from the hotplate and allowed to cool. The watch glass was washed with deionized water and samples were diluted to 50 mL. A portion was filtered for analysis by inductively coupled plasma—optical emission spectroscopy (ICP-OES). Measured isotopes investigated were: ^23^Na, ^24^Mg, ^31^P, ^39^K, ^44^Ca, ^55^Mn, ^56^Fe, ^59^Co, ^63^Cu, ^66^Zn, ^78^Se, ^95^Mo, ^111^Cd, ^202^Hg, ^208^Pb. Stock solutions (1000 mg/L) of the element standards were used for preparing calibration curves and as internal standard^2^. The ICP-OES analysis was performed using an Agilent 7700X spectrometer (Agilent Technologies) equipped with nickel sampler cone and a MicroMist nebulizer with a reference to APHA 3120B methodologies^51^. ICP-OES analysis duration was 3.0 min, one point data was obtained with three replicates and 100 sweeps per replicate.

### SDS-PAGE profiling of protein isolate

For SDS-PAGE analysis, protein isolates were prepared by micellisation. Here, lupin flours were extracted twice by adding 0.2% NaOH (at 1:5 ratio of flour to NaOH) for 14h with occasional shaking and then centrifuged at 3000 × *g* for 30 min. The combined supernatants were then diluted with distilled water (10 fold) and left to stand for 18 h at 4 °C. The precipitated protein isolates were separated by centrifugation (as above), freeze dried and collected^34,52^. The SDS-PAGE analysis of the protein isolates were done by standard procedure using the Laemmli buffer system. For protein samples, a final concentration of 2 mg/mL was obtained with 10% glycerol (v/v), 1% SDS (w/v), 1 mM EDTA, 10 mM Tris-Cl pH 6.8 and dithiothreitol (DTT, 0.5% w/v) as the reducing agent. 2 × concentrate of sample buffer was prepared, denaturation was done by heating at 95 °C for 5 minutes and the insoluble materials were removed by centrifugation at 3000 × *g* for 30 min. The electrophoresis was carried out in a 13% gel of polyacrylamide (w/v). Molecular weight marker from Bio-Rad was used and the protein bands were stained with Coomassie Brilliant Blue R-250^53,54^.

### Pasting properties

Pasting properties of the lupin flour samples were determined using a Rapid Visco Analyzer (RVA-S4, Newport Scientific, Narrabeen, Australia) using the standard method of the instrument. Flour sample (3.5 g, weight was adjusted on the basis of 14% moisture content) was added to an RVA aluminium canister containing 25 mL distilled water and blended well by its paddle at room temperature. It was then heated at 50 °C for 1 min. Finally, the slurry was heated from 50 to 95 °C at a rate of 12°C/min and held at 95 °C for 2.5 min. It was then cooled from 95 to 50 °C at a rate of 12 °C/min and held at 50 °C for 2 min. The mixing paddle speed was maintained at 160 rpm. Parameters including peak viscosity, trough, peak time and final viscosity were recorded^36,55^.

### Textural properties

The flour pastes obtained after heating and cooking in the RVA canister (from the RVA test) were sealed with parafilm, and kept at 4°C for 24h to form a gel, which was then evaluated for gel textural properties using a TA.XT Plus Texture Analyzer (Stable Micro Systems, Surrey, UK). A penetration test was performed with at a pretest speed of 2 mm/s, test speed of 3 mm/s and a post-test speed of 10 mm/s to a distance of 10 mm with a cylindrical stainless steel probe (50 mm diameter). The slope of the force versus distance curve was used as hardness (g/mm), surface area of force versus distance was taken as work required to penetrate the gel (g.mm) and maximum force was taken as peak force (g). Measurements were made in triplicates^36,56^.

### Thermal properties

Thermal properties were assessed using a Perkin Elmer DSC-7 Differential Scanning Calorimeter. The analysis was carried out at temperatures between 30 and 120 °C, at a rate of 10 °C/min, on a 1:3 (w/w) lupin flour-water mixture sample. Before heating in the DSC, samples were hermetically sealed and allowed to stand at room temperature for 1 hour. Two protein denaturation peaks were observed and analyzed using the software supplied by the manufacturer and onset (T_o_), peak (T_p_), and conclusion (T_c_) temperatures were determined. The enthalpy of protein denaturation (ΔH) was calculated and expressed as J/g of dry lupin flour^8,36^.

### Functional properties of lupin cultivars

The following functional properties of lupin cultivars’ flours were assayed in the present work.

#### Swelling power

Swelling power of lupin flour samples were determined by the method described by Yu et al. with minor modifications^57^. Lupin flour (1 g) was mixed with 10 mL distilled water in a centrifuge tube (15 mL) and heated at 80 °C for 30 min with occasional shaking in a water bath. The mixture was centrifuged at 112 × *g* for 15 min and the supernatant layer was decanted. The paste (swelled flour) was then weighed.

$$\mathrm{Swelling\,\, power }(\mathrm{g}/\mathrm{g})=\frac{\mathrm{Weight\,\, of\,\, swelled\,\, flour}}{\mathrm{Weight\,\,   of\,\,  dry\,\, flour}}$$

### Water and oil absorption capacity

Water and oil absorption capacities were determined by the method described by Rodriguez-Ambriz et al. with minor modifications^58^. Lupin flour (0.5 g) was taken in a 15 mL centrifuge tube and either 5 mL of distilled water or canola oil was added to it. The test tube was agitated for 2 min with a vortex shaker for complete dissolution. The test tube was then immerged in a water bath at a temperature of 28 °C for 30 min. Centrifugation for 20 min at 72 × *g* enabled the separation of the supernatant layer, which was decanted and the volume of water or free oil (supernatant) measured.

$$\mathrm{Water}/\mathrm{oil\,\, absorption\,\, capacity }(\mathrm{mL}/\mathrm{g})=\frac{\mathrm{Volume\,\, of\,\, water}/\mathrm{oil\,\,  added}-\mathrm{Volume\,\, of \,\, free \,\, water}/\mathrm{oil}}{\mathrm{Weight\,\, of\,\, sample\,\, taken}}$$

### Emulsifying capacity and stability

Emulsifying capacity and emulsion stability was measured by the method of Cano-Medina et al. with minor modifications^46^. Lupin flour (1 g) and 12.5 mL of distilled water was blended for 2 min in order to form a homogenous mixture using a Microtron laboratory mixer. Canola oil (7 mL) was added to the mixture and blended further for 1 min. The prepared emulsion was centrifuged at 1600 × *g* for 10 min. For measuring emulsion stability, the prepared emulsion was heated in a water bath at 85 °C for 30 min. It was then cooled to room temperature. After that it was centrifuged at 1600 × *g* for 10 min. The emulsifying capacity and emulsion stability of the lupin cultivars were calculated by using the following formula.

$$\mathrm{Emulsion \,\, capacity}/\mathrm{stability }(\%)=\frac{\mathrm{Height \,\, of\,\,  emulsified\,\,  layer}}{\mathrm{Height\,\,  of \,\, total \,\, contents\,\,  of \,\, tube}} \times 100$$

### Creaming stability of the emulsion

Creaming stability of the emulsion was determined by the method described by Tornberg with modifications^45^. After homogenization, 10 mL of the emulsion was transferred into a 15 mL centrifuge tube, which was tightly sealed and kept at ambient temperature. It was periodically checked for the separation of emulsion. After 12 h it was observed that the emulsion had separated into an opaque layer at the top and a turbid layer at bottom. Creaming stability of the emulsion was calculated using the following formula.

$$\mathrm{Creaming \,\,  stability }(\%)=\frac{\mathrm{Height\,\,  of\,\,  serum \,\, layer}}{\mathrm{Height\,\,  of \,\, the\,\,  turbid \,\, layer}} \times 100$$

#### Foaming capacity and stability

Foaming capacity and foam stability was measured by the method of Cano-Medina et al. with minor modifications^46^. Firstly, 1 g flour and 50 mL 0.01 M Na_2_HPO_4_-NaH_2_PO_4_ buffer (pH 7) were placed in a beaker. The mixture was hydrated for about 15 min and then blended for around 2 min with the Microtron laboratory mixer. The total volume was recorded. Foam capacity was expressed as foam expansion at 0 min and foam stability was expressed as foam expansion after 60 min. Foam capacity was calculated from the following equation.

$$\mathrm{Foam  \,\, capacity }(\mathrm{\%})=\frac{\mathrm{Final \,\, volume}-\mathrm{Initial \,\, volume}}{\mathrm{Initial\,\,  volume}} \times 100$$

### Statistical analysis

Statistical evaluations of test results were done by one-way ANOVA (*p* < 0.05). Pair-wise comparison of mean values were performed using a Post-Hoc Tukey test. SPSS statistical analysis software of IBM Corporation, New York, USA (version 16.0) was used for analyzing the data and assessing the results^59^.

## Supplementary Information

Supplementary Information.

## Data Availability

All data generated during this study are included within our manuscript and in the supplementary materials’ file. The original (raw)/processed data necessary for the reproduction of these findings are available upon request from the corresponding authors.
