# Supplementary material for: Comparative assessment of nutritional, thermal, rheological and functional properties of nine Australian lupin cultivars
Source: Sci Rep. 2021 Nov 2;11:21515. doi: 10.1038/s41598-021-00838-x (PMC8564527; doi:10.1038/s41598-021-00838-x)
Supplement: Supplementary file 1 — Supplementary Information. [file 41598_2021_838_MOESM1_ESM.pdf]

Comparative assessment of nutritional, thermal, rheological and functional properties of nine Australian lupin cultivars

Kishor Mazumder[1,2,*], **Biswajit Biswas**[1], **Philip G Kerr**[2,*], **Christopher Blanchard**[2], **Afia Nabila**[3], **Mimi Golder**[4], **Mohammad Gulzarul Aziz**[5] **& Asgar Farahnaky**[2,6]

[1] Department of Pharmacy, Faculty of Biological Science and Technology, Jashore University of Science and Technology, Jashore - 7408, Bangladesh
[2] School of Biomedical Sciences, Charles Sturt University, Wagga Wagga, New South Wales - 2650, Australia
[3] Department of Pharmacy, University of Science and Technology Chittagong, Chittagong - 4202, Bangladesh
[4] Pharmacy Discipline, Life Science School, Khulna University, Khulna - 9208, Bangladesh
[5] Department of Food Technology and Rural Industries, Bangladesh Agricultural University, Mymensingh - 2202, Bangladesh
[6] Biosciences and Food Technology, School of Science, RMIT University, Melbourne - 3083, Australia

*Correspondence to: kmazumder@just.edu.bd, pkerr@csu.edu.au

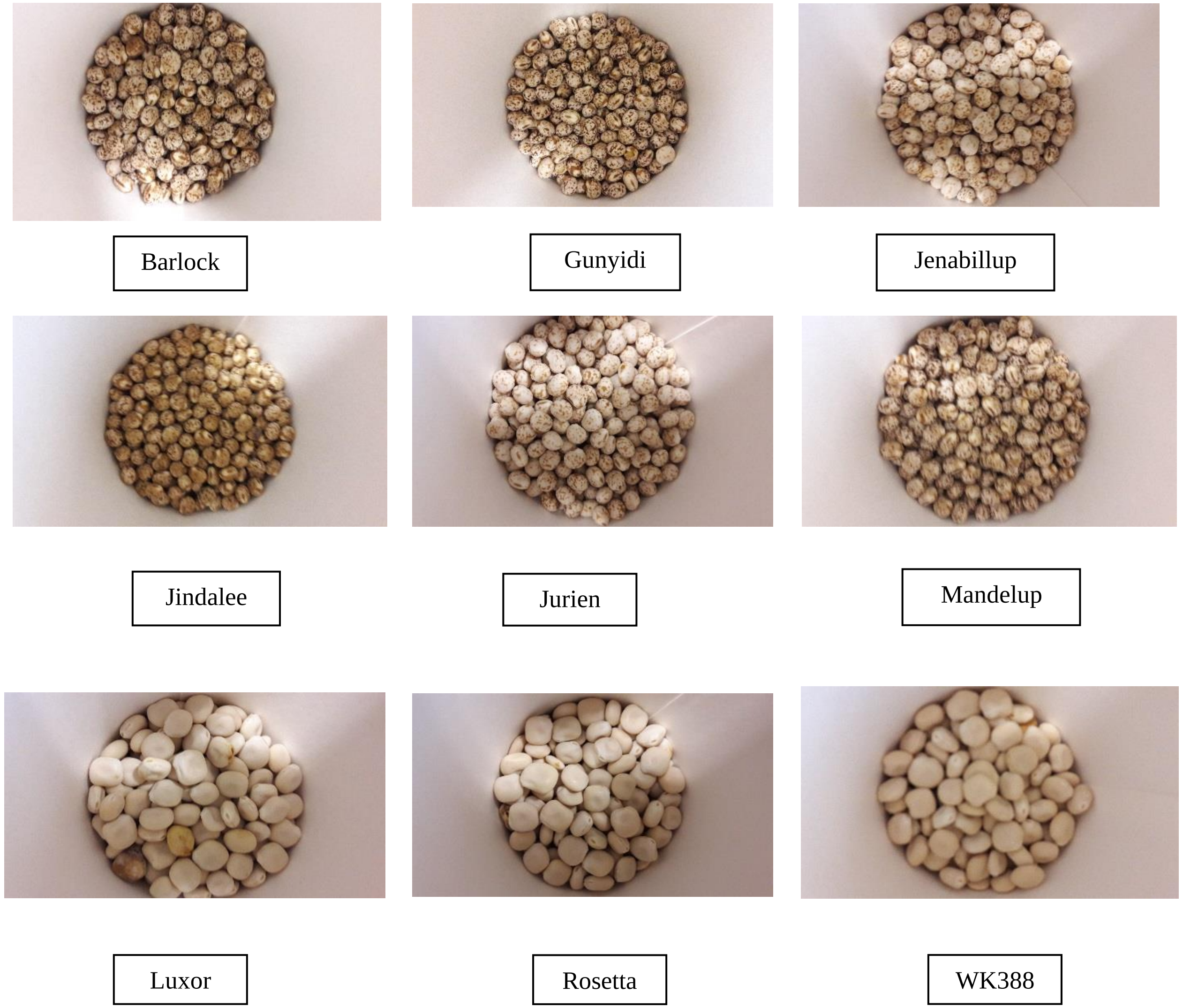

Supplementary Figure S1. Nine Australian lupin cultivars used in the present work

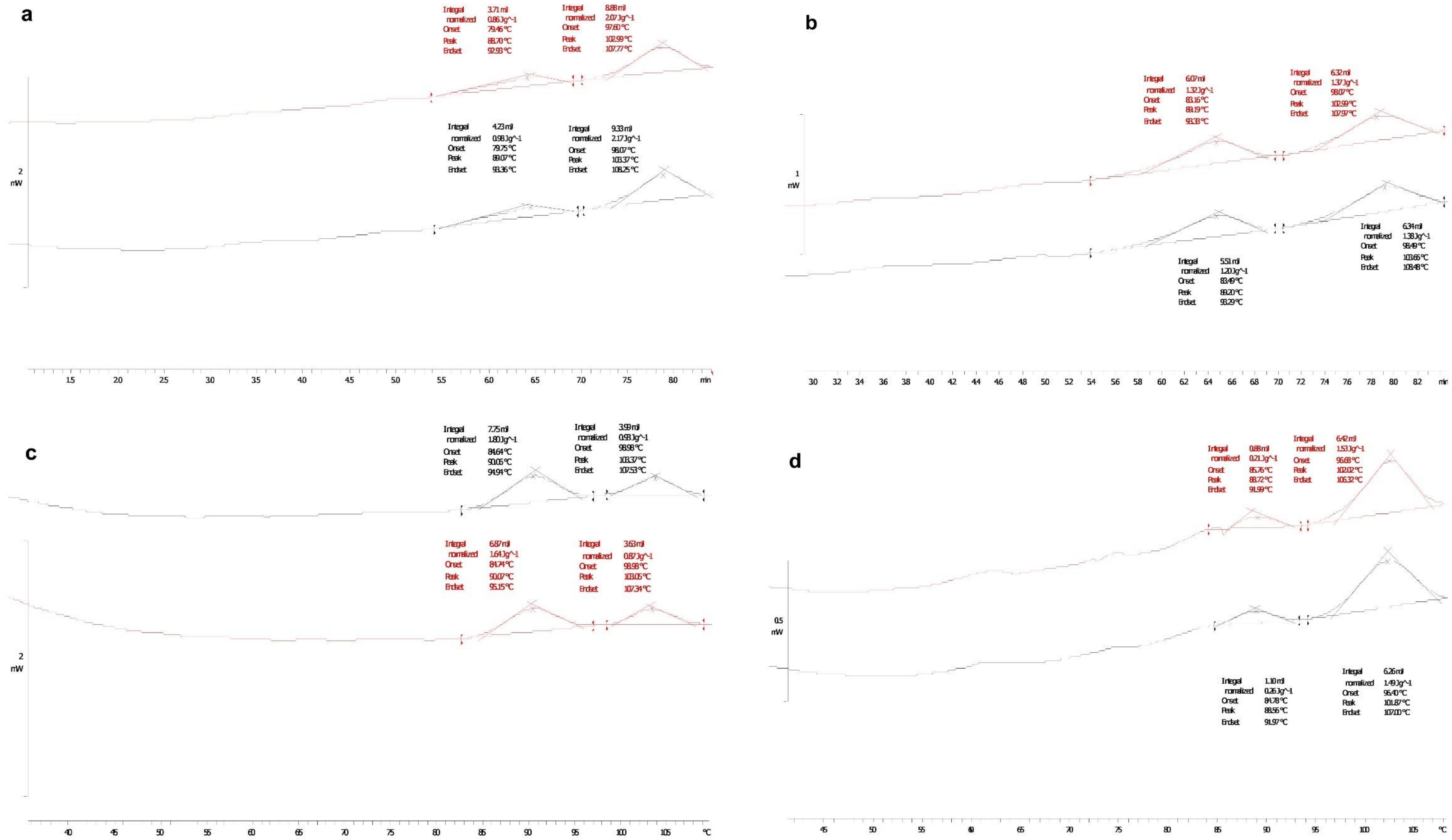
a
Integral 3.71 mJ normalized 0.86 Jg^-1 Onset 79.46 °C Peak 88.70 °C Endset 92.93 °C
Integral 8.88 mJ normalized 2.07 Jg^-1 Onset 97.60 °C Peak 102.99 °C Endset 107.77 °C
Integral 4.23 mJ normalized 0.98 Jg^-1 Onset 79.75 °C Peak 89.07 °C Endset 93.36 °C
Integral 9.33 mJ normalized 2.17 Jg^-1 Onset 98.07 °C Peak 103.37 °C Endset 108.25 °C
2 mW
15 20 25 30 35 40 45 50 55 60 65 70 75 80 min
b
Integral 6.07 mJ normalized 1.32 Jg^-1 Onset 83.16 °C Peak 89.19 °C Endset 93.33 °C
Integral 6.32 mJ normalized 1.37 Jg^-1 Onset 98.07 °C Peak 102.99 °C Endset 107.97 °C
Integral 6.34 mJ normalized 1.38 Jg^-1 Onset 98.49 °C Peak 103.65 °C Endset 108.48 °C
Integral 5.51 mJ normalized 1.20 Jg^-1 Onset 83.49 °C Peak 89.20 °C Endset 93.29 °C
1 mW
30 32 34 36 38 40 42 44 46 48 50 52 54 56 58 60 62 64 66 68 70 72 74 76 78 80 82 min
c
Integral 7.75 mJ normalized 1.80 Jg^-1 Onset 84.64 °C Peak 90.05 °C Endset 94.94 °C
Integral 3.99 mJ normalized 0.93 Jg^-1 Onset 98.98 °C Peak 103.37 °C Endset 107.53 °C
Integral 6.87 mJ normalized 1.64 Jg^-1 Onset 84.74 °C Peak 90.07 °C Endset 95.15 °C
Integral 3.63 mJ normalized 0.87 Jg^-1 Onset 98.98 °C Peak 103.05 °C Endset 107.34 °C
2 mW
40 45 50 55 60 65 70 75 80 85 90 95 100 105 °C
d
Integral 0.88 mJ normalized 0.21 Jg^-1 Onset 85.76 °C Peak 88.72 °C Endset 91.99 °C
Integral 6.42 mJ normalized 1.53 Jg^-1 Onset 96.68 °C Peak 102.02 °C Endset 106.32 °C
0.5 mW
Integral 1.10 mJ normalized 0.26 Jg^-1 Onset 84.78 °C Peak 88.56 °C Endset 91.97 °C
Integral 6.26 mJ normalized 1.49 Jg^-1 Onset 96.40 °C Peak 101.87 °C Endset 107.00 °C
45 50 55 60 65 70 75 80 85 90 95 100 105 °C

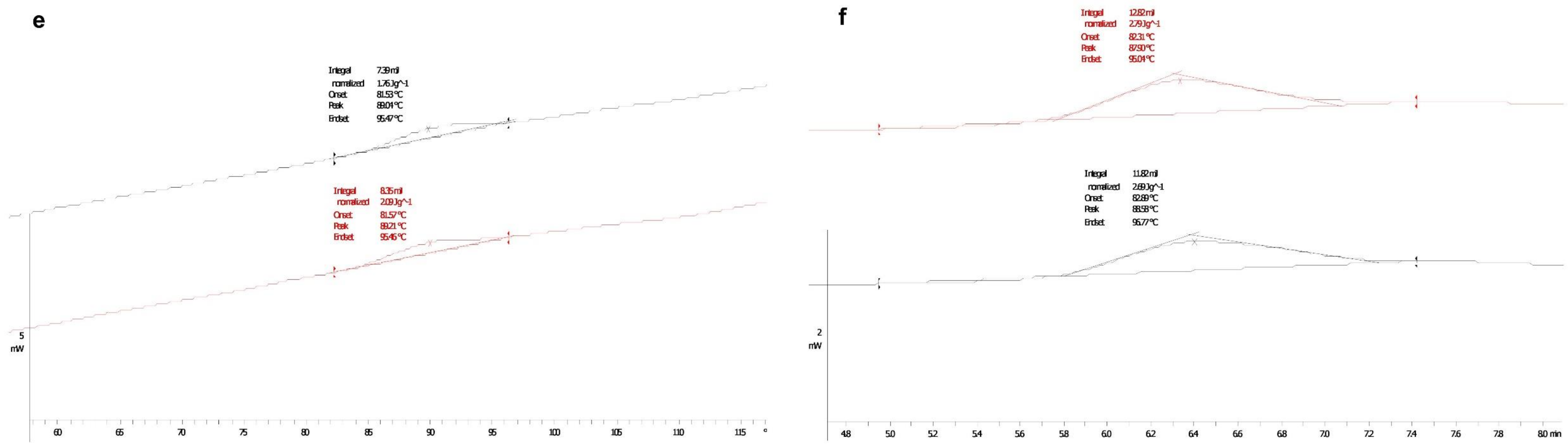

Supplementary Figure S2. Thermograms of proteins of some lupin flours; where, **a**: Gunyidi, **b:** Jenabillup, **c:** Jindalee, **d:** Mandelup, **e:** Luxor, **f:** Rosetta. The two peaks in the DSC thermogram indicate the presence of two major protein classes: peak 1 represents the vicilins (β-conglutin) and peak 2 represents the legumins (α-conglutin). The red lines represent DSC thermograms of protein isolates while the black lines represent the thermograms of lupin flours.

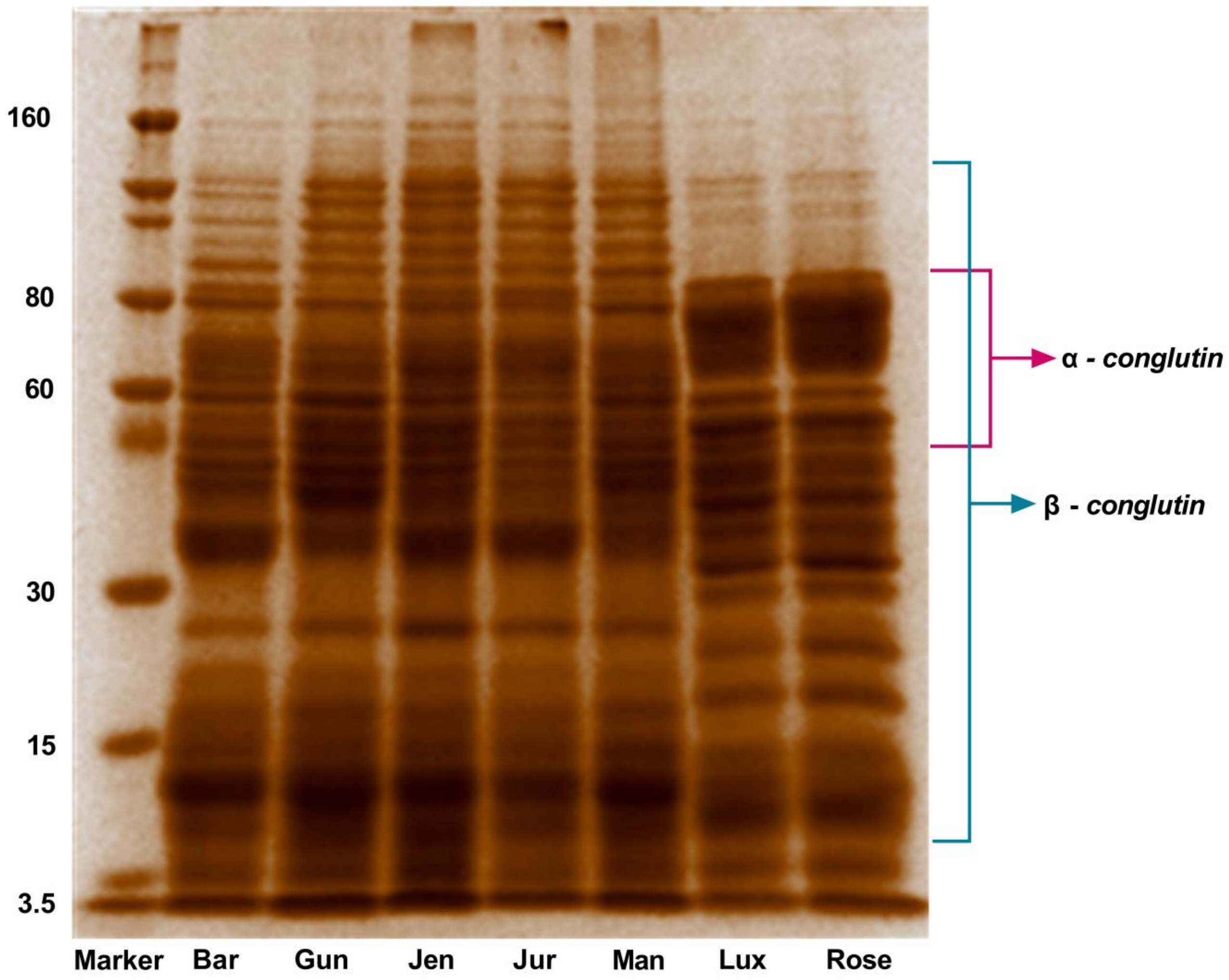

Supplementary Figure S3. Electrophoretic pattern of major protein isolates (β-, α- and γ -conglutin) of lupin cultivars, separated by SDS-PAGE analysis. Here, Bar is Barlock, Gun is Gunyidi, Jen is Jenabillup, Jur is Jurien, Man is Mandelup, Lux is Luxor and Rose is Rosetta.

Lupin Cultivars	Initial Weight (gm)	Kernal Weight (gm)	%	Seed Coat Weight (gm)	%	Intermediate (gm)	%
**Barlock**	533.8	362.1	67.83	126	23.60	45.7	8.56
**Gunyidi**	524	353.3	67.42	129.4	24.69	41.3	7.88
**Jenabillup**	504	345.8	68.61	115.06	22.83	43.16	8.56
**Jindalee**	525.4	360.0	68.52	121.3	23.09	44.1	8.39
**Jurin**	509.7	354.7	69.59	116.9	22.94	38.1	7.47
**Mandelup**	519.9	354.3	68.15	123.6	23.77	42	8.08
**Luxor**	510	364.1	71.39	83.9	16.45	62	12.16
**Rosetta**	508.8	374.6	73.62	78.4	15.41	55.8	10.97
**WK388**	514.5	368.4	71.60	78	15.16	68.1	13.24

Supplementary Table S1. Kernel and seed coat contents of different cultivars obtained after dehulling of lupin seeds.

Cultivars	Peak 1 (mPa-s)	Peak 2 (mPa-s)	Trough (mPa-s)	Peak 3 (mPa-s)	Peak 1 Time (sec)	Peak 2 Time (sec)	Peak 3 Time (sec)	Final Viscosity (mPa-s)
**Barlock**	588.5 ± 3.54 ^{c,f,g,h,i}	578.5 ± 37.48 ^{b,c,d,g,h,i}	512 ± 8.49 ^{c,i}		9.65 ± 0.03 ^{g,h,i}	12.25 ± 0.04		1405 ± 22.63 ^{d}
**Gunyidi**	590 ± 4.24 ^{c,f,g,h,i}	464.5 ± 13.44 ^{a,g,h,i}	411 ± 8.49		9.69 ± 0.02 ^{g,h,i}	11.83 ± 0.84		1190 ± 19.80
**Jenabillup**	506.5 ± 11.32 ^{a,b,d,e,f,g,h,i}	379.5 ± 4.95 ^{a,f,g,h,i}	352.5 ± 3.54 ^{a}	316.2 ± 0.23 ^{e}	9.9 ± 0.14 ^{g,h,i}	12.2 ± 0.07	16.2 ± 0.23	1028 ± 31.11 ^{g}
**Jindalee**	586.5 ± 10.61 ^{c,f,g,h,i}	427 ± 9.9 ^{a,g,h,i}	403.5 ± 7.78		9.54 ± 0.04 ^{g,h,i}	11.54 ± 0.05		863 ± 19.8 ^{a,g}
**Jurien**	561.5 ± 34.64 ^{c,f,g,h,i}	477 ± 18.38 ^{g,h,i}	438 ± 15.56	426.03 ± 0.0 ^{c}	9.72 ± 0.16 ^{g,h,i}	12.38 ± 0.0	16.0 ± 0.0	1090.5 ± 34.65
**Mandelup**	688 ± 7.07 ^{a,b,c,d,e,g,h,i}	521 ± 19.8 ^{c,g,h,i}	446.5 ± 41.72 ^{h}		9.92 ± 0.07 ^{g,h,i}	12.59 ± 0.12		1251.5 ± 40.31
**Luxor**	175 ± 0.0 ^{a,b,c,d,e,f,h,i}	1036 ± 32.53 $^{a.b,c,d,e,f,h,i}$	414 ± 31.11		4.95 ± 0.07 ^{a,b,c,d,e,f}	12.12 ± 0.05		1593 ± 347.9 ^{c,d}
**Rosetta**	122.5 ± 0.71 ^{a,b,c,d,e,f,g,i}	900 ± 41.01 ^{a,b,c,d,e,f,g}	327.5 ± 61.52 ^{a,f}		4.92 ± 0.16 ^{a,b,c,d,e,f}	11.95 ± 0.04		1138 ± 183.85
**WK388**	147 ± 4.24 ^{a,b,c,d,e,f,g,h}	860 ± 41.01 ^{a,b,c,d,e,f,g,h}	390 ± 33.94 ^{a}		4.85 ± 0.14 ^{a,b,c,d,e,f}	12.04 ± 0.02		1078.5 ± 21.92

Supplementary Table S2. RVA profile of different lupin cultivars. Data are means of three replicates with standard deviations (SD). Data within the same column with different superscripts are significantly different, pair-wise comparison by Post Hoc Tukey test (^{a} $P < 0.05$ vs. Barlock; ^{b} $P < 0.05$ vs. Gunyidi; ^{c} $P < 0.05$ vs Jenabillup; ^{d} $P < 0.05$ vs. Jindalee; ^{e} $P < 0.05$ vs Jurien; ^{f} $P < 0.05$ vs. Mandelup; ^{g} $P < 0.05$ vs Luxor; ^{h} $P < 0.05$ vs Rosetta; ^{i} $P < 0.05$ vs WK388).

Cultivars	Hardness (g/mm)	Work (g.mm)	Peak Force (g)
**Barlock**	34.17 ± 9.88 [g,h]	373.41 ± 18 [b,c,d,e,f,g,h,i]	376.92 ± 22.62 [b,c,d,e,g,h,i]
**Gunyidi**	44.04 ± 0.4 [h,i]	2612.72 ± 80.65 [a,c,d,e,f,g,h,i]	442.04 ± 7.17 [a,c,e,f,g,h,i]
**Jenabillup**	32.36 ± 3.09 [g,h]	2195.45 ± 72 [a,b,d,e,f,g,h,i]	413.01 ± 80.73 [a,b,e,f,g,i]
**Jindalee**	33.20 ± 44 [g,h]	2649.84 ± 25 [a,b,c,e,f,g,h,i]	425.56 ± 70.31 [a,e,f,g,i]
**Jurin**	41.24 ± 6.31 [g,i]	2825.86 ± 323.66 [a,b,c,d,f,g,h,i]	474.13 ± 87.99 [a,b,c,d,f,g,h,i]
**Mandelup**	30.08 ± 1.23 [g,h]	2950.11 ± 227.93 [a,b,c,d,e,g,h,i]	374.16 ± 47.61 [b,c,d,e,g,h,i]
**Luxor**	50.62 ± 11.72 [a,c,d,f,i]	3269.63 ± 286.47 [a,b,c,d,e,f,h,i]	506.59 ± 46.70 [a,b,c,d,e,f,h,i]
**Rosetta**	63.68 ± 4.79 [a,b,c,d,e,f,i]	2088.84 ± 42.46 [a,b,c,d,e,f,g,i]	407.01 ± 4.95 [a,b,e,f,g,i]
**WK388**	26.71 ± 3.25 [b,e,g,h]	1321.62 ± 186.28 [a,b,c,d,e,f,g,h]	220.12 ± 25.96 [a,b,c,d,e,f,g,h]

Supplementary Table S3. Texture analysis of different lupin cultivars. Data are means of three replicates with standard deviations (SD). Data within the same column with different superscripts are significantly different, pair-wise comparison by Post Hoc Tukey test ([a] $P < 0.05$ vs. Barlock; [b] $P < 0.05$ vs. Gunyidi; [c] $P < 0.05$ vs Jenabillup; [d] $P < 0.05$ vs. Jindalee; [e] $P < 0.05$ vs Jurin; [f] $P < 0.05$ vs. Mandelup; [g] $P < 0.05$ vs Luxor; [h] $P < 0.05$ vs Rosetta; [i] $P < 0.05$ vs WK388).
